# Supplementary material for: Nonspecific cleavages arising from reconstitution of trypsin under mildly acidic conditions
Source: PLoS One. 2020 Jul 28;15(7):e0236740. doi: 10.1371/journal.pone.0236740 (PMC7386593; doi:10.1371/journal.pone.0236740)
Supplement: S1 File — (DOCX) [file pone.0236740.s001.docx]

**The amino acid sequence of NISTmAb**

>NISTmAb Heavy Chain with Lys

QVTLRESGPALVKPTQTLTLTCTFSGFSLSTAGMSVGWIRQPPGKALEWLADIWWDDKKHYNPSLKDRLTISKDTSKNQV

VLKVTNMDPADTATYYCARDMIFNFYFDVWGQGTTVTVSSASTKGPSVFPLAPSSKSTSGGTAALGCLVKDYFPEPVTVS

WNSGALTSGVHTFPAVLQSSGLYSLSSVVTVPSSSLGTQTYICNVNHKPSNTKVDKRVEPKSCDKTHTCPPCPAPELLGG

PSVFLFPPKPKDTLMISRTPEVTCVVVDVSHEDPEVKFNWYVDGVEVHNAKTKPREEQYNSTYRVVSVLTVLHQDWLNGK

EYKCKVSNKALPAPIEKTISKAKGQPREPQVYTLPPSREEMTKNQVSLTCLVKGFYPSDIAVEWESNGQPENNYKTTPPV

LDSDGSFFLYSKLTVDKSRWQQGNVFSCSVMHEALHNHYTQKSLSLSPGK

>NISTmAb Light Chain

DIQMTQSPSTLSASVGDRVTITCSASSRVGYMHWYQQKPGKAPKLLIYDTSKLASGVPSRFSGSGSGTEFTLTISSLQPD

DFATYYCFQGSGYPFTFGGGTKVEIKRTVAAPSVFIFPPSDEQLKSGTASVVCLLNNFYPREAKVQWKVDNALQSGNSQE

SVTEQDSKDSTYSLSSTLTLSKADYEKHKVYACEVTHQGLSSPVTKSFNRGEC
