# Supplementary material for: Nonspecific cleavages arising from reconstitution of trypsin under mildly acidic conditions
Source: PLoS One. 2020 Jul 28;15(7):e0236740. doi: 10.1371/journal.pone.0236740 (PMC7386593; doi:10.1371/journal.pone.0236740)
Supplement: S3 File — (DOCX) [file pone.0236740.s003.docx]

**Intact mass measurements**

Trypsin-1 samples were reconstituted in HPLC-grade water and 50 mM acetic acid, respectively, for 4 hours at room temperature, before submitting for intact mass measurements. In brief, 2 μg of sample was loaded onto an Acquity BEH300 C4 column and eluted using a 20-min gradient running from 95% mobile phase A + 5% mobile phase B to 5% mobile phase A + 95% mobile phase B. The mobile phase A consisted of 0.1% formic acid in water, mobile phase B consisted of 0.1% formic acid in acetonitrile. A Thermo Scientific Q-Exactive HF-X mass spectrometer operated in full-scan mode with resolving power at 15k and *m/z* range of 600-4000 was used for mass detection. The data analysis and deconvolution of obtained intact mass spectra were performed using Protein Metrics Intact Mass software.


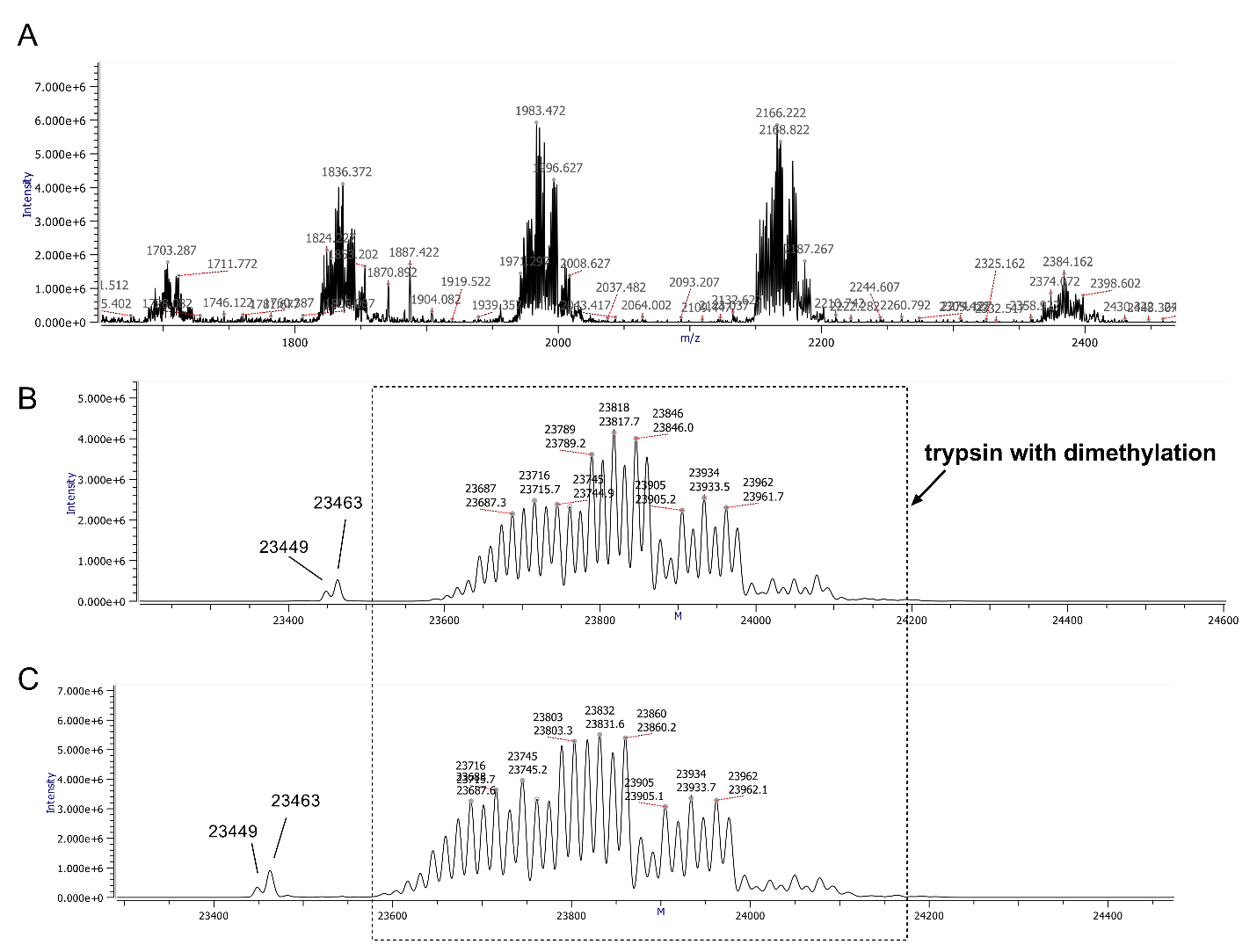


The intact mass measurements outcome. (A) raw mass spectra of charge envelop showing that, within each charge state, the trypsin exhibited numerous peaks, indicating a highly heterogenous mass distribution; (B) deconvoluted mass spectra corresponding to Trypsin-1 reconstituted in 50 mM acetic acid. Although the intact mass of Trypsin-1 were detected (23463 Da), the predominant species were the trypsin with high extent of dimethylation (peaks in box), the mass shift between any adjacent two peaks were 14 Da, corresponding to the difference of a modification of methylation; (C) deconvoluted mass spectra corresponding to Trypsin-1 reconstituted in water. Similarly, the major species detected were the trypsin with dimethylation modifications, the intact Trypsin-1 (with mass 23463 Da) only accounted for minor amount.

The mass profiles obtained for the two trypsin samples were highly comparable, however, trypsin peptide bond cleavages which render +18 Da shifts were unattainable from these spectra.
