# Supplementary material for: Nonspecific cleavages arising from reconstitution of trypsin under mildly acidic conditions
Source: PLoS One. 2020 Jul 28;15(7):e0236740. doi: 10.1371/journal.pone.0236740 (PMC7386593; doi:10.1371/journal.pone.0236740)
Supplement: S4 File — (DOCX) [file pone.0236740.s004.docx]

**Cation-Exchange Chromatography (CEX)**

The cation exchange chromatography (CEX) analysis of trypsin was conducted on a Waters BioResolve SCX column (3 μm 4.5x50 mm) using a phosphate pH gradient. The gradient was 40 mM NaH_2_PO_4_ to 40 mM NaH_2_PO_4_/Na_2_HPO_4_ 50/50 (v/v) in 20 min with a flow rate of 0.3 mL/min. The column load was 10 μg. The separation was achieved at room temperature and monitored at 280 nm.

**
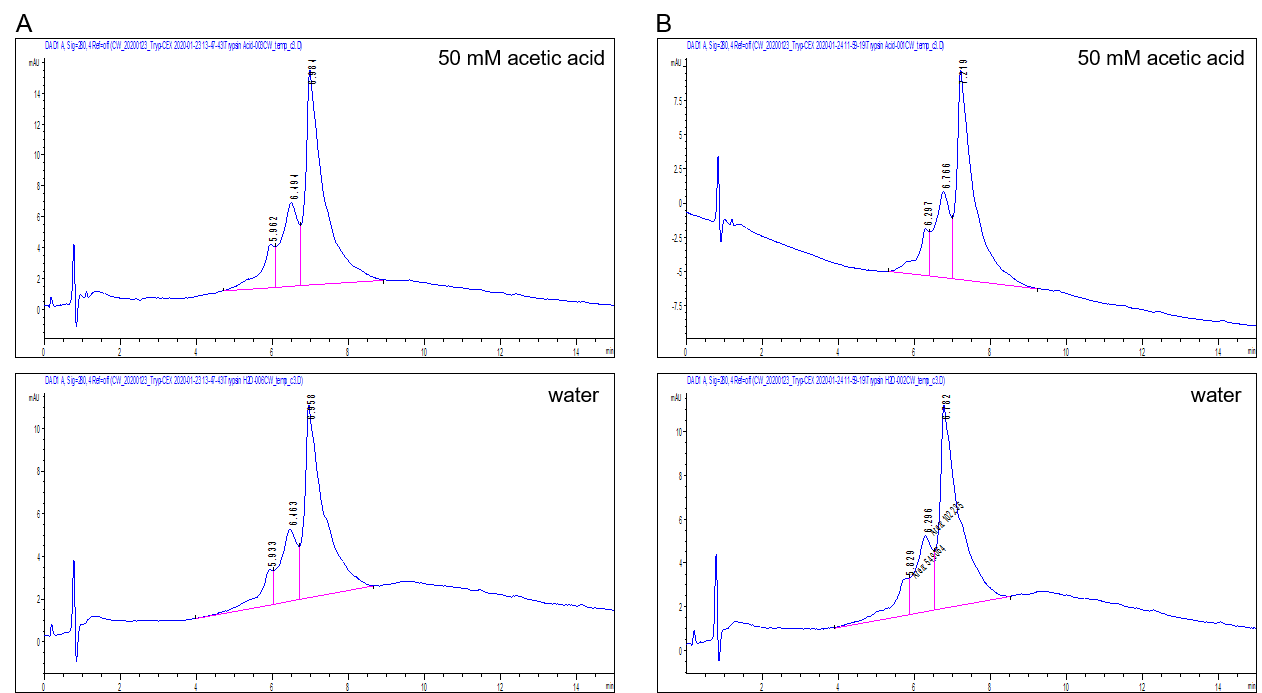
**

| **A (t0)** | | | |
| --- | --- | --- | --- |
| **50 mM acetic acid** | **#** | **Time (min)** | **Area%** |
|  | **1** | **5.96** | **10.48** |
|  | **2** | **6.49** | **22.88** |
|  | **3** | **6.98** | **66.64** |
| **water** | **1** | **5.93** | **9.98** |
|  | **2** | **6.46** | **21.59** |
|  | **3** | **6.96** | **68.43** |

| **B (t=24h)** | | | |
| --- | --- | --- | --- |
| **50 mM acetic acid** | **#** | **Time (min)** | **Area%** |
|  | **1** | **6.30** | **10.53** |
|  | **2** | **6.77** | **23.23** |
|  | **3** | **7.22** | **66.24** |
| **water** | **1** | **5.83** | **11.14** |
|  | **2** | **6.30** | **20.74** |
|  | **3** | **6.78** | **68.12** |

The chromatography profiles we obtained were similar to the previously reported ion-exchange chromatography of trypsin autolyze [1]. Figure A corresponds to the UV profiles of CEX separation of Trypsin-1 reconstituted in 50 mM acetic acid (upper panel) and HPLC-grade water (lower panel) immediately before chromatography (t0). Figure B corresponds to the profiles of Trypsin-1 reconstituted in 50 mM acetic acid (upper panel) and HPLC-grade water (lower panel) for 24 hours prior to CEX separation. Quantitation of relative peak areas (Area%) demonstrated highly comparable profiles among these conditions, indicating that no new forms of trypsins were being generated by reconstituting in 50 mM acetic acid, and the observed increase of nontryptic activities upon acidic reconstitution may not be due to increased amount of pseudotrypsin.

**Reference**

1. Perutka Z, Sebela M. Pseudotrypsin: A Little-Known Trypsin Proteoform. Molecules. 2018;23(10). Epub 2018/10/17. doi: 10.3390/molecules23102637. PubMed PMID: 30322187; PubMed Central PMCID: PMCPMC6222510.
