# Supplementary material for: Nonspecific cleavages arising from reconstitution of trypsin under mildly acidic conditions
Source: PLoS One. 2020 Jul 28;15(7):e0236740. doi: 10.1371/journal.pone.0236740 (PMC7386593; doi:10.1371/journal.pone.0236740)
Supplement: S1 Fig — UV chromatograms overlay corresponding to 6 biological replicates of monoclonal antibody A (mAb-A) digested with Trypsin-1 reconstituted in (A) 50 mM acetic acid (B) HPLC water, respectively, to demonstrate the reproducibility of sample preparation and analysis. UV peaks of peptide H34 (heavy chain 373–393) and H14γ (heavy chain153-185) were annotated; (C) comparison of the XIC areas of peptide H14γ and H34, in between the two different trypsin reconstitution conditions (50 mM acetic acid vs. water), show significant increase of peptide H14γ, as generated from trypsin nonspecific cleavage, in the acetic acid reconstitution condition; the XIC areas of the fully-tryptic peptide H34 remained unchanged. T test P values were used to indicate statistical significance of the differences (*** for P ≤ 0.001, and ns for P > 0.05). The error bars correspond to the ± SE of the 6 replicates measurements. (DOCX) [file pone.0236740.s005.docx]

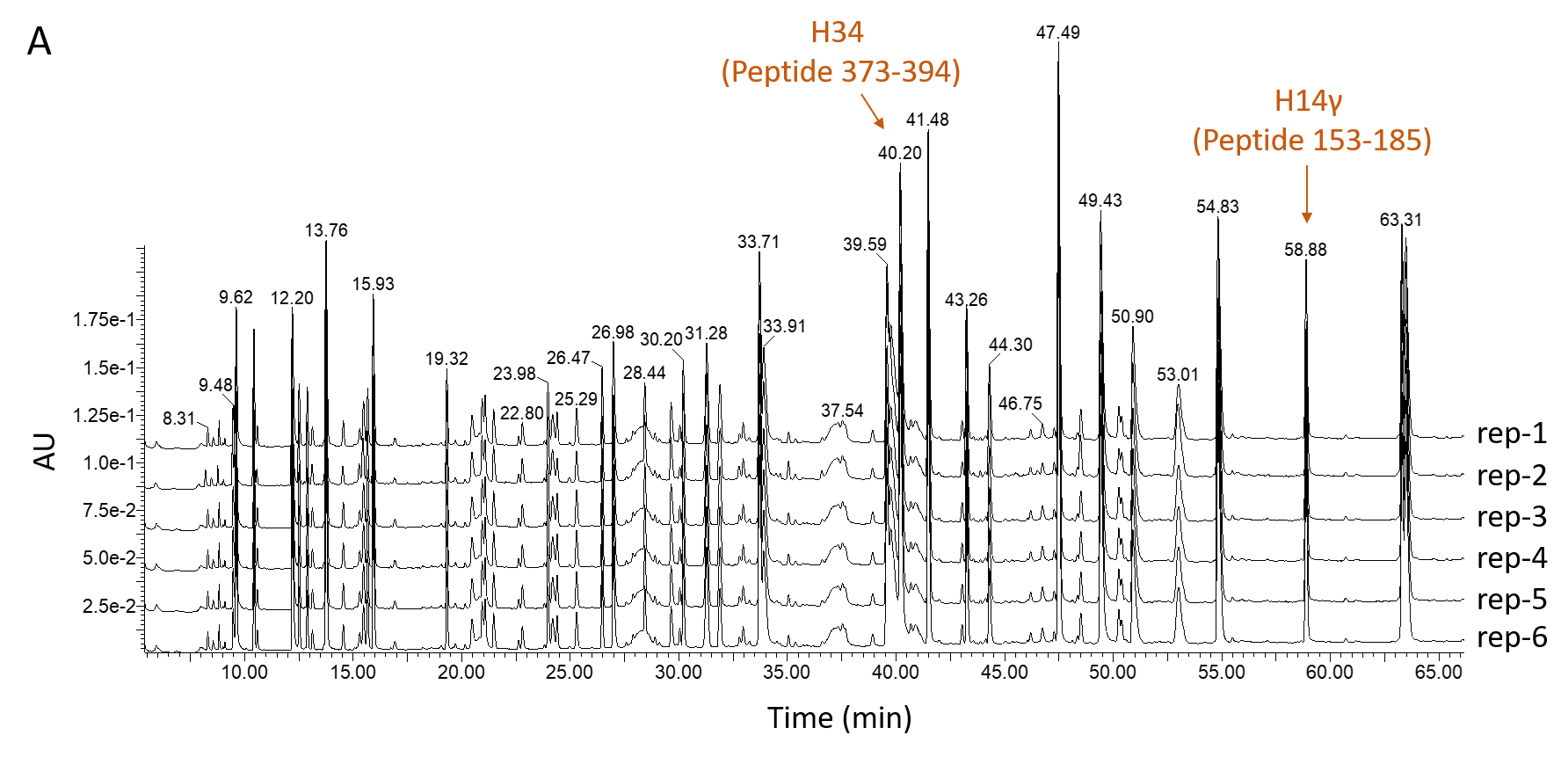


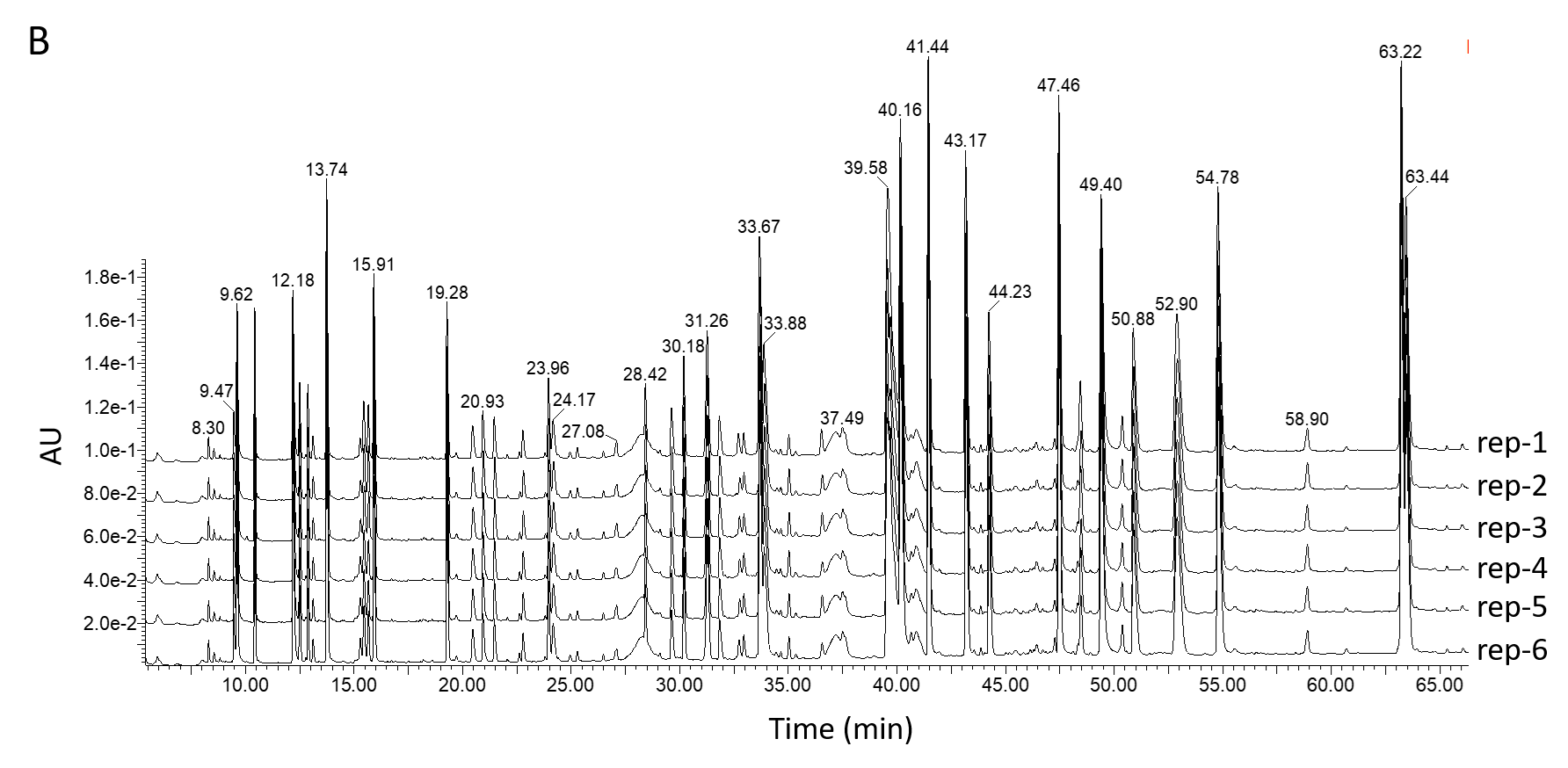


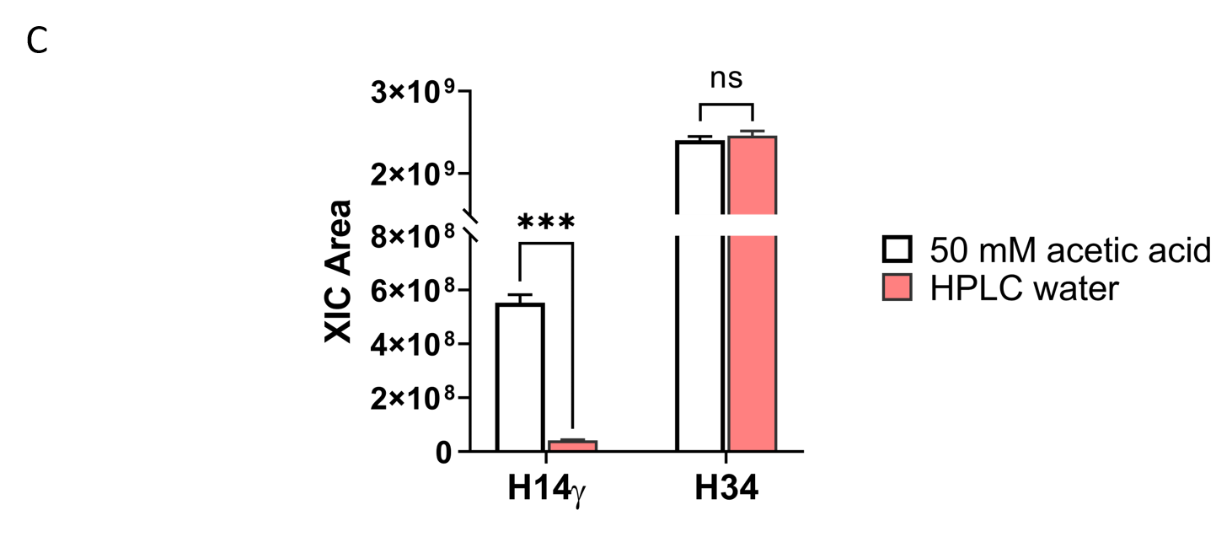


**Fig S1.** UV chromatograms overlay corresponding to 6 biological replicates of monoclonal antibody A (mAb-A) digested with Trypsin-1 reconstituted in (A) 50 mM acetic acid (B) HPLC water, respectively, to demonstrate the reproducibility of sample preparation and analysis. UV peaks of peptide H34 (heavy chain 373-393) and H14γ (heavy chain153-185) were annotated; (C) comparison of the XIC areas of peptide H14γ and H34, in between the two different trypsin reconstitution conditions (50 mM acetic acid *vs.* water), show significant increase of peptide H14γ, as generated from trypsin nonspecific cleavage, in the acetic acid reconstitution condition; the XIC areas of the fully-tryptic peptide H34 remained unchanged. T test P values were used to indicate statistical significance of the differences (*** for P ≤ 0.001, and ns for P > 0.05). The error bars correspond to the $\pm$ SE of the 6 replicates measurements.
