# Supplementary material for: Nonspecific cleavages arising from reconstitution of trypsin under mildly acidic conditions
Source: PLoS One. 2020 Jul 28;15(7):e0236740. doi: 10.1371/journal.pone.0236740 (PMC7386593; doi:10.1371/journal.pone.0236740)
Supplement: S2 Fig — (A) Overlay of UV chromatograms of trypsin-digested mAb-A, using trypsin reconstituted in 1 mM HCl, 50 mM acetic acid, and high-performance liquid chromatography (HPLC)–grade water. The peak profiles of the digestion with trypsins reconstituted in acid were highly similar to, but different from, those with trypsin reconstituted in water. Nonspecific cleavages were significantly higher with acetic acid reconstitution. The five dashed-line boxes indicate selected regions in which additional peaks corresponding to nonspecific cleavages arose; (B) zoomed-view of the five dashed-line boxes across different trypsin reconstitution conditions. (DOCX) [file pone.0236740.s006.docx]

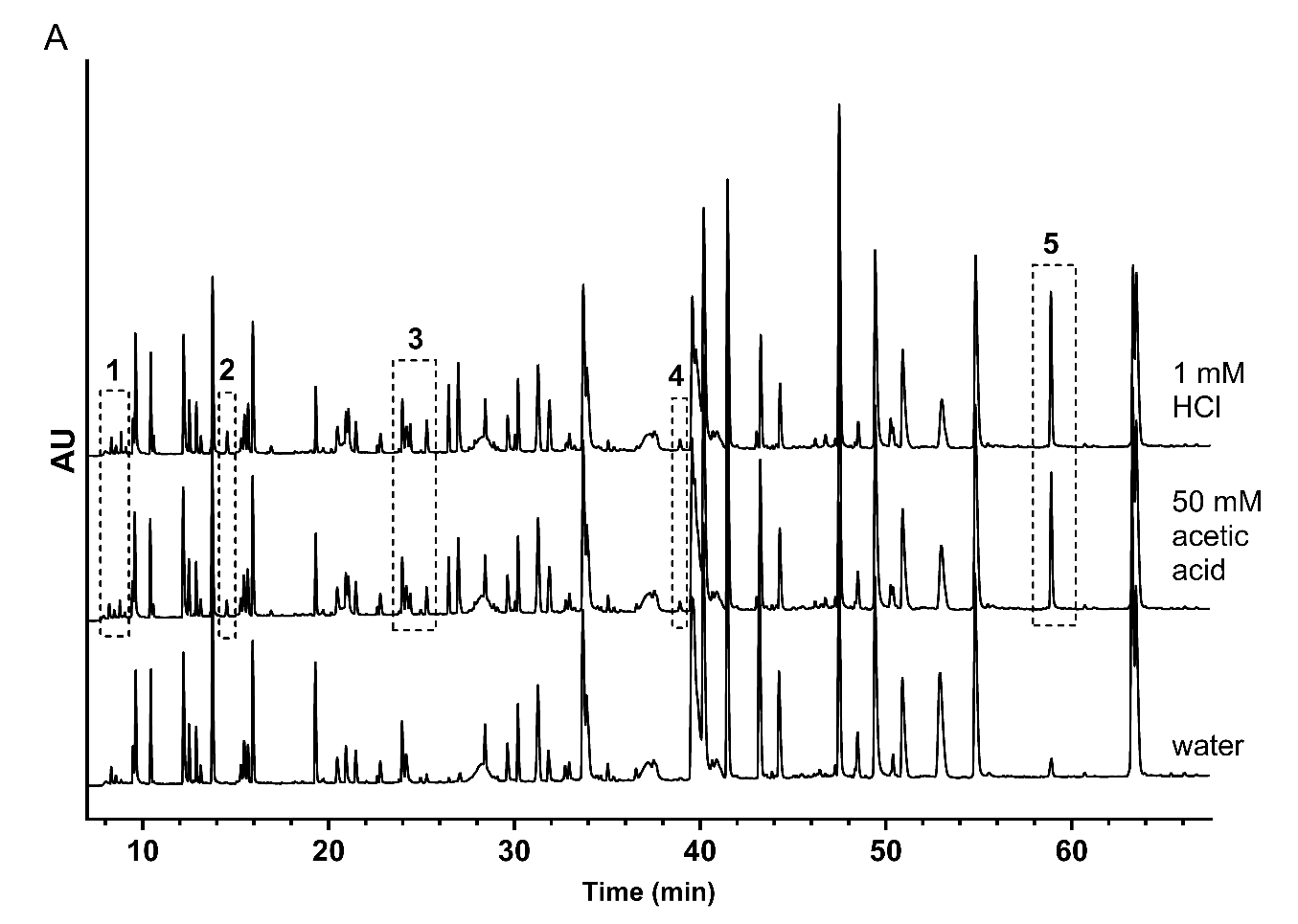


**
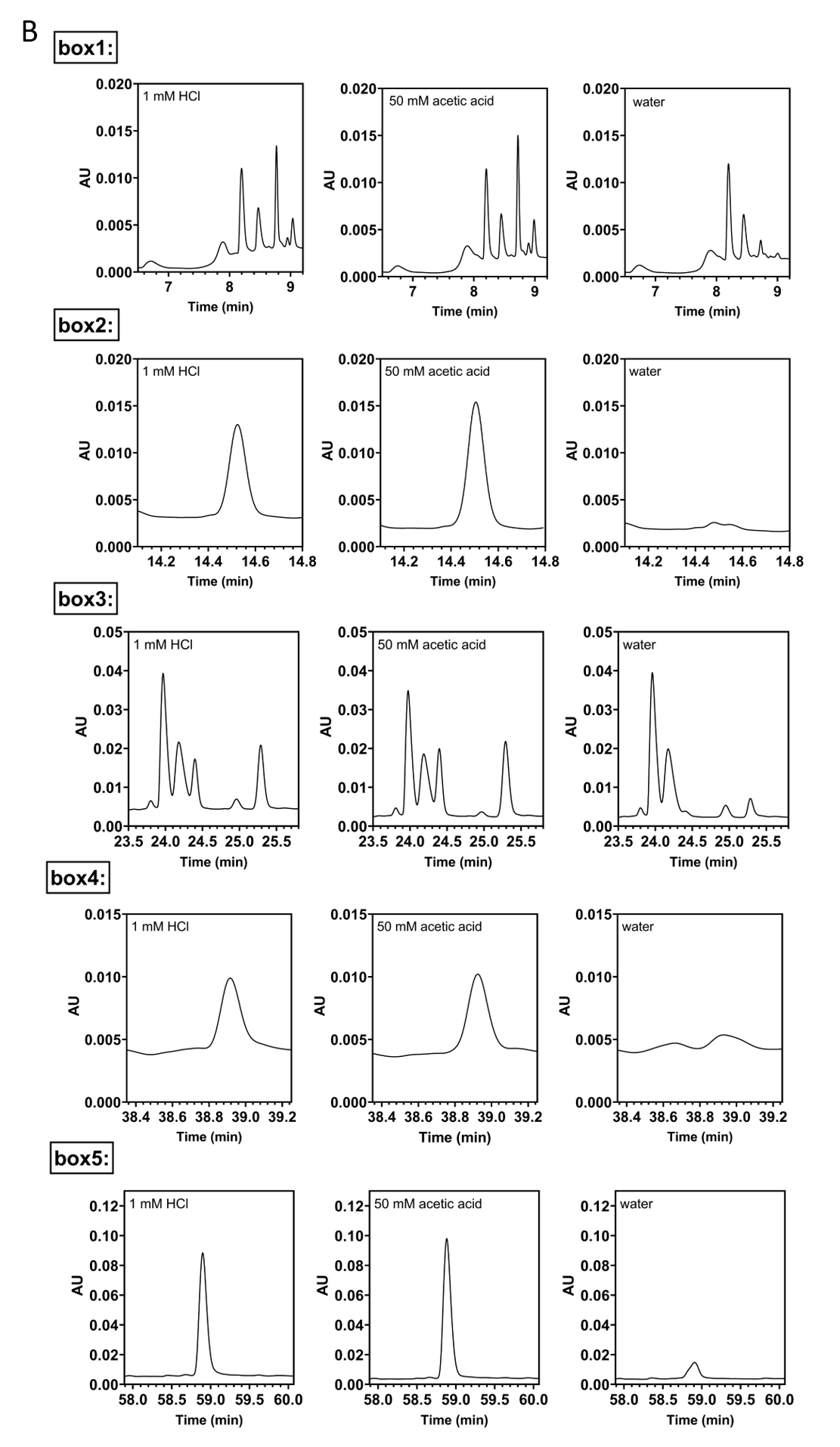
**

**Fig S2.** (A) Overlay of UV chromatograms of trypsin-digested mAb-A, using trypsin reconstituted in 1 mM HCl, 50 mM acetic acid, and high-performance liquid chromatography (HPLC)–grade water. The peak profiles of the digestion with trypsins reconstituted in acid were highly similar to, but different from, those with trypsin reconstituted in water. Nonspecific cleavages were significantly higher with acetic acid reconstitution. The five dashed-line boxes indicate selected regions in which additional peaks corresponding to nonspecific cleavages arose; (B) zoomed-view of the five dashed-line boxes across different trypsin reconstitution conditions.
