# Supplementary material for: Nonspecific cleavages arising from reconstitution of trypsin under mildly acidic conditions
Source: PLoS One. 2020 Jul 28;15(7):e0236740. doi: 10.1371/journal.pone.0236740 (PMC7386593; doi:10.1371/journal.pone.0236740)
Supplement: S3 Fig — (A) Total ion current chromatograms corresponding to the tryptic digestion of a 17-kDa protein with trypsin reconstituted in HPLC-grade water (upper panel) and 1 mM HCl (lower panel). The two visible new peaks (shaded in blue) were identified as peptides 30–48 and 91–110 from nontryptic cleavages at W48 and Tyr110, respectively. (B) Extracted ion chromatograms of peptides 30–48 and 91–110, showing the dramatic increase in nontryptic cleavages that occurred when 1 mM HCl was used for trypsin reconstitution. (DOCX) [file pone.0236740.s007.docx]

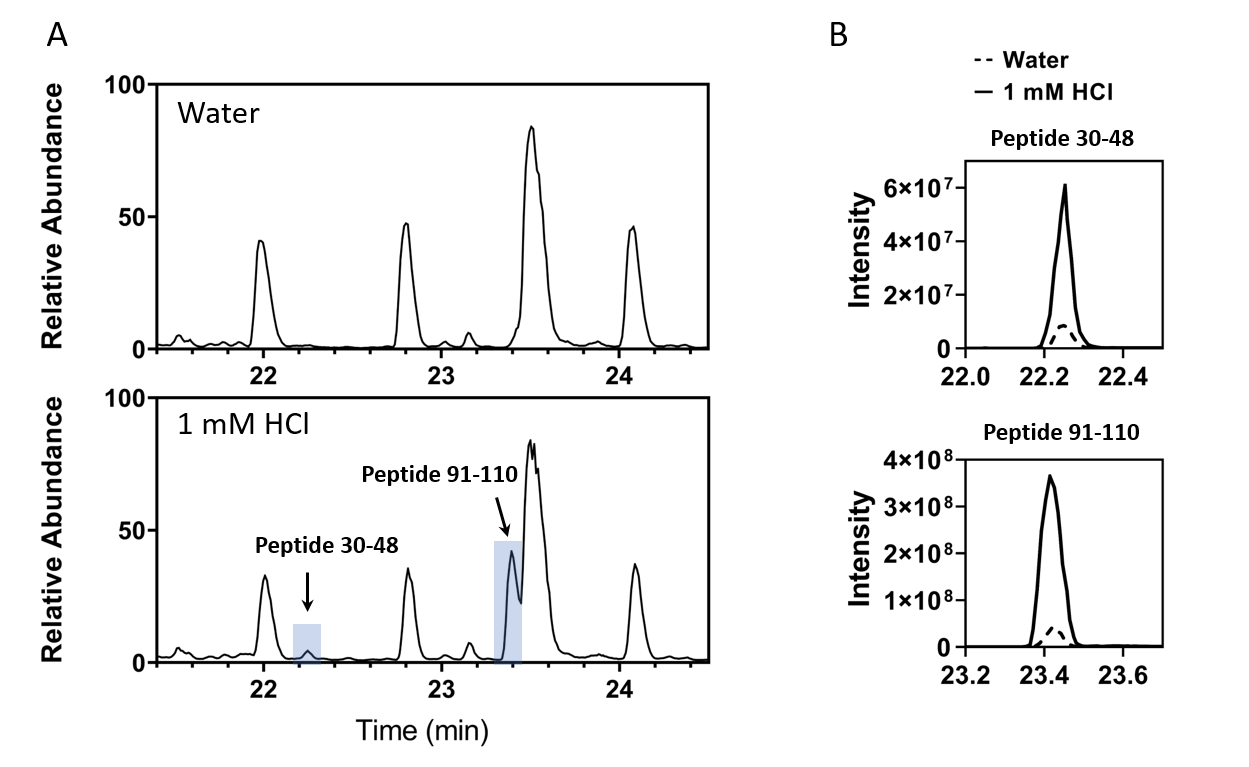


**Fig S3.** (A) Total ion current chromatograms corresponding to the tryptic digestion of a 17-kDa protein with trypsin reconstituted in HPLC-grade water (upper panel) and 1 mM HCl (lower panel). The two visible new peaks (shaded in blue) were identified as peptides 30–48 and 91–110 from nontryptic cleavages at W48 and Tyr110, respectively. (B) Extracted ion chromatograms of peptides 30–48 and 91–110, showing the dramatic increase in nontryptic cleavages that occurred when 1 mM HCl was used for trypsin reconstitution.
