# Supplementary material for: Nonspecific cleavages arising from reconstitution of trypsin under mildly acidic conditions
Source: PLoS One. 2020 Jul 28;15(7):e0236740. doi: 10.1371/journal.pone.0236740 (PMC7386593; doi:10.1371/journal.pone.0236740)
Supplement: S4 Fig — (A) New peak detection analysis designated 121 species as “new,” based on the predefined peak selection criteria. The apex retention time of each species versus the corresponding monoisotopic m/z is plotted. (B) Mass distribution of 58 peptides deconvoluted from the 121 species. Approximately 50% of peptides had masses ranging from 1,200 to 1,800 Da. (DOCX) [file pone.0236740.s008.docx]

**
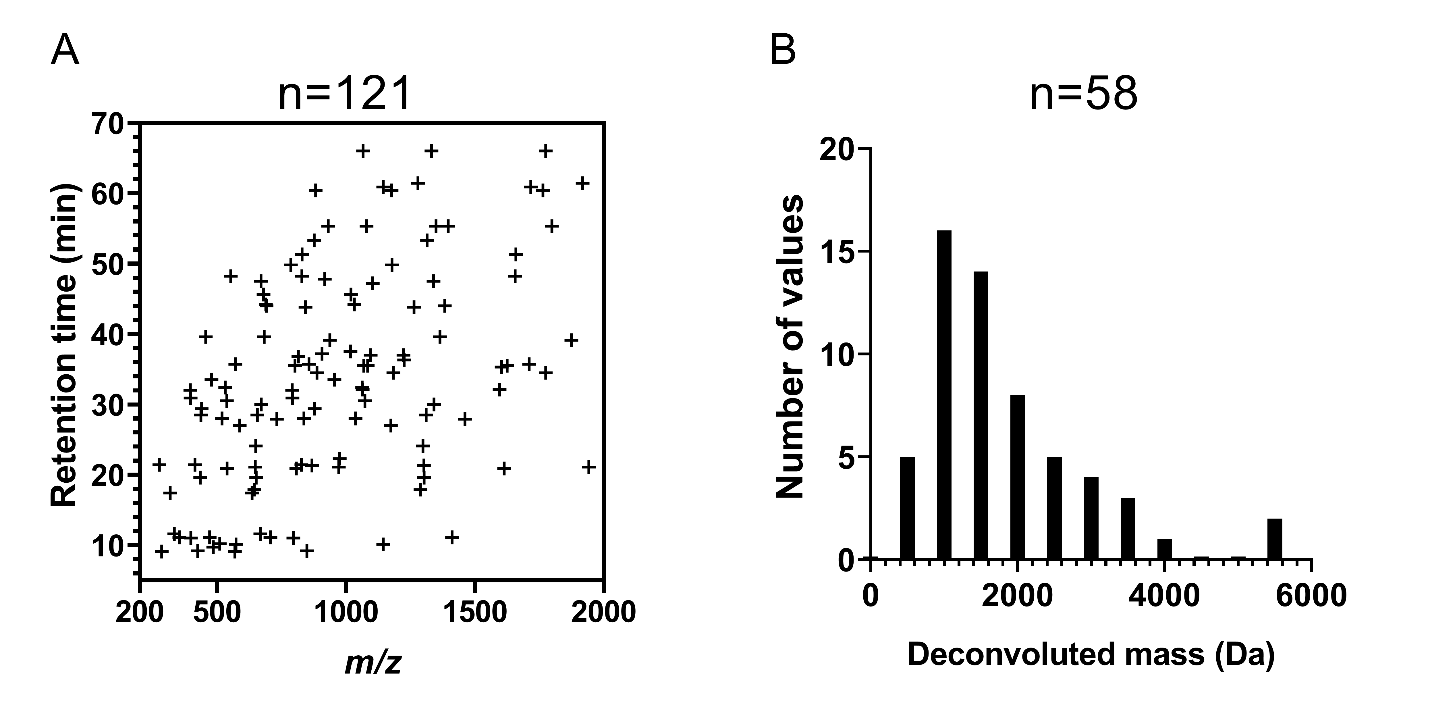
**

**Fig S4.** (A) New peak detection analysis designated 121 species as “new,” based on the predefined peak selection criteria. The apex retention time of each species versus the corresponding monoisotopic m/z is plotted. (B) Mass distribution of 58 peptides deconvoluted from the 121 species. Approximately 50% of peptides had masses ranging from 1,200 to 1,800 Da.
