# Supplementary material for: Nonspecific cleavages arising from reconstitution of trypsin under mildly acidic conditions
Source: PLoS One. 2020 Jul 28;15(7):e0236740. doi: 10.1371/journal.pone.0236740 (PMC7386593; doi:10.1371/journal.pone.0236740)
Supplement: S5 Fig — Although all peptides showed fold-change values of a minimum of 3, nontryptic peptides demonstrated more significant fold-change values than did semitryptic peptides. Statistical analysis was performed using the Student t test. (DOCX) [file pone.0236740.s009.docx]

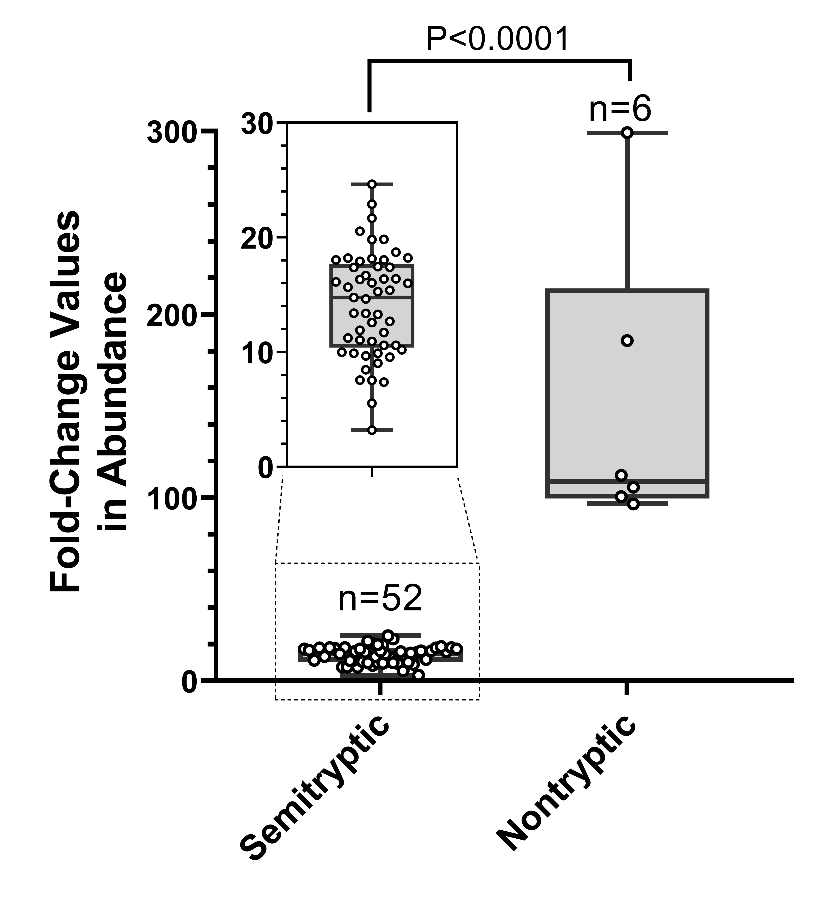


**Fig S5.** Box plot showing the fold-change values in abundance for all identified semitryptic and nontryptic peptides. Although all peptides showed fold-change values of a minimum of 3, nontryptic peptides demonstrated more significant fold-change values than did semitryptic peptides. Statistical analysis was performed using the Student *t* test.
