# Supplementary material for: Nonspecific cleavages arising from reconstitution of trypsin under mildly acidic conditions
Source: PLoS One. 2020 Jul 28;15(7):e0236740. doi: 10.1371/journal.pone.0236740 (PMC7386593; doi:10.1371/journal.pone.0236740)
Supplement: S6 Fig — Assessment of the extent of missed cleavages (A, B) showed that the levels of missed cleavages ranged from 4% to 7% for all conditions. Assessment of the extent of trypsin autolysis (C, D) showed that the levels of autolysis ranged from 2% to 4% for all conditions. (DOCX) [file pone.0236740.s010.docx]

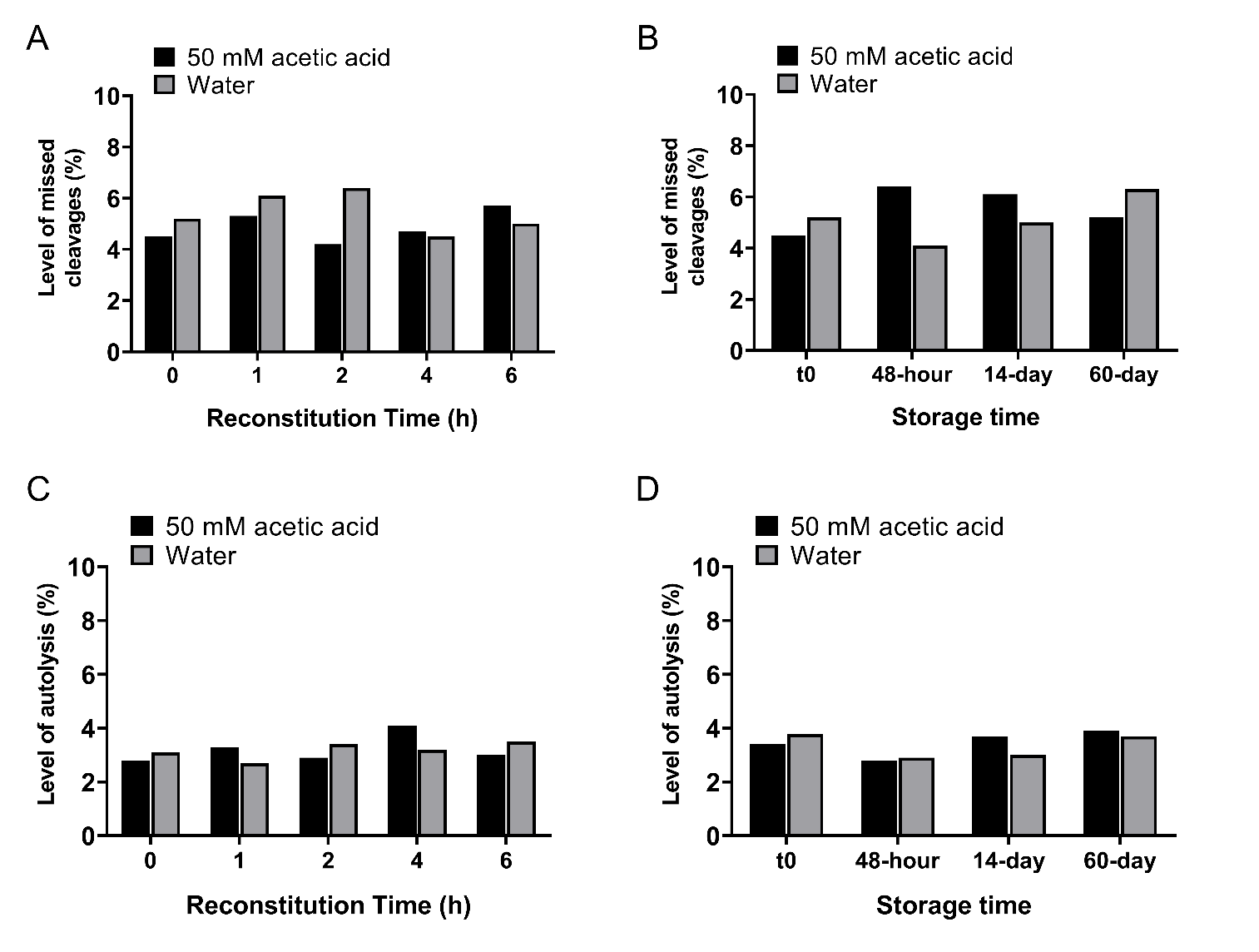


**Fig S6.** Assessment of the extent of missed cleavages (A, B) showed that the levels of missed cleavages ranged from 4% to 7% for all conditions. Assessment of the extent of trypsin autolysis (C, D) showed that the levels of autolysis ranged from 2% to 4% for all conditions.
